# An intuitionistic approach to scoring DNA sequences against transcription factor binding site motifs

**DOI:** 10.1186/1471-2105-11-551

**Published:** 2010-11-08

**Authors:** Fernando Garcia-Alcalde, Armando Blanco, Adrian J Shepherd

**Affiliations:** 1 Bionformatics and Genomics Department, Centro de Investigación Príncipe Felipe (CIPF), Valencia 46013, Spain; 2 Department of Computer Science and Artificial Intelligence, University of Granada, Granada 18071, Spain; 3 Department of Biological Sciences and Institute of Structural and Molecular Biology, Birkbeck College, University of London, Malet Street, London WC1E 7HX, UK

## Abstract

**Background:**

Transcription factors (TFs) control transcription by binding to specific regions of DNA called transcription factor binding sites (TFBSs). The identification of TFBSs is a crucial problem in computational biology and includes the subtask of predicting the location of known TFBS motifs in a given DNA sequence. It has previously been shown that, when scoring matches to known TFBS motifs, interdependencies between positions within a motif should be taken into account. However, this remains a challenging task owing to the fact that sequences similar to those of known TFBSs can occur by chance with a relatively high frequency. Here we present a new method for matching sequences to TFBS motifs based on intuitionistic fuzzy sets (IFS) theory, an approach that has been shown to be particularly appropriate for tackling problems that embody a high degree of uncertainty.

**Results:**

We propose *SC_intuit_*, a new scoring method for measuring sequence-motif affinity based on IFS theory. Unlike existing methods that consider dependencies between positions, *SC_intuit _*is designed to prevent overestimation of less conserved positions of TFBSs. For a given pair of bases, *SC_intuit _*is computed not only as a function of their combined probability of occurrence, but also taking into account the individual importance of each single base at its corresponding position. We used *SC_intuit _*to identify known TFBSs in DNA sequences. Our method provides excellent results when dealing with both synthetic and real data, outperforming the sensitivity and the specificity of two existing methods in all the experiments we performed.

**Conclusions:**

The results show that *SC_intuit _*improves the prediction quality for TFs of the existing approaches without compromising sensitivity. In addition, we show how *SC_intuit _*can be successfully applied to real research problems. In this study the reliability of the IFS theory for motif discovery tasks is proven.

## Background

Cells control the abundance of proteins by means of diverse mechanisms. One such mechanism is the regulation of transcription, which is a continuous process whereby many factors combine to ensure appropriate rates of protein synthesis. Understanding such complex processes is one of the main objectives in computational biology. In its early stages, transcription is controlled, among other mechanisms, by the binding of proteins called transcription factors (TFs) to specific regions of a given chromosome called transcription factor binding sites (TFBSs). These interactions between proteins and DNA usually take place upstream from the gene, close to the transcription start site (TSS), in the so-called promoter region of the gene.

One of the biggest issues in identifying TFBSs is that a single binding protein can bind to different DNA sequences. Related DNA sequences to which the same TF can bind are grouped together into a TFBS motif. The identification of TFBSs within a given set of DNA sequences is an active area of research. In this context there exist two main approaches: *i) *the *de novo *discovery of motifs, and *ii) *the detection of TFBSs using motifs that are already known.

*De novo *methods aim to find significant sub-sequence patterns within a set of TFBS sequences. Some of the most popular approaches are MEME [1], Gibbs sampling [2], AlignACE [3], and more recently PRIORITY and Trawler [4,5]. For a review see [6].

Detection methods, on the other hand, focus on inferring new TFBSs from known binding motifs. Early detection methods assumed independence between positions within a putative TFBS sequence, e.g. in Patser [7] and ConSite [8]. However, it is now well established that this assumption is wrong [9-11], and some methods that consider position dependency for modeling and finding TFBSs using advanced HMM and Bayesian models have appeared [12,13]. Likewise, two recent detection methods have been developed that take into account interdependencies between TFBS positions. Tomovic and Oakeley proposed a method that incorporates a measure of positional interdependence into the overall score [14]. More recently, Zare-Mirakabad et al. developed a method based on joint information content and mutual information [15]. In this method, positional dependencies are taken into account by considering all pairwise combinations of positions (see the Methods section for more information).

The fact that TFBS sequences are usually very short means that the same or very similar sequences tend to occur by chance at a relatively high frequency. Consequently one of the main goals in the prediction of TFBSs is to reduce the false positive rate without compromising sensitivity. Methods that take into account positional dependencies tend to be significantly more effective at meeting this challenge. However, there remains room for improvement. As we will show in the Results section, existing methods have some drawbacks, such as overlearning of the training data, arbitrary threshold selection for testing dependencies, etc. The purpose of the work presented here is to provide a new method for measuring sequence-motif affinity that improves on existing approaches.

Zadeh proposed fuzzy sets theory to mathematically model the imprecision inherent in certains concepts [16]. Briefly, fuzzy sets theory allows an object to partially belong to a set with a membership degree between 0 and 1. Classical set theory is a special case of its fuzzy counterpart in which membership and certainty degrees are restricted to either 0 or 1. Atanassov proposed intuitionistic fuzzy sets (IFS) theory as an extension of the fuzzy sets theory [17]. IFSs generalize the notion of a fuzzy set representing uncertainty with respect to both the degree of membership (*μ*) and non-membership (*ν*) of a set by allowing that the sum *μ *+ *ν *≤ 1.

Owing to the fact that IFSs are capable of modelling the uncertainty present in real-life situations, they have been widely applied during the past decades to a variety of problems (see the Methods section). In recent years, it has been seen that the inherent uncertainty and noise that characterize biological data cannot always be modeled sufficiently well using probabilistic approaches and that, as a consequence, alternative approaches to modelling this uncertainty may be required [18-21]. In addition to the usual problems of missing values and noisy data associated with biological data, there exist some additional hidden factors that affect binding affinities in the context of sequence-motif scoring, e.g. cooperative binding and chromatin structure [22]. Furthermore, the described motifs are subject to change as new experiments confirm new binding sites. In this work we make use of IFS theory to formally model the uncertainty associated with the problem of scoring DNA sequences against TFBS motifs.

## Results

### Case studies

First, we wanted to show the ability of our proposed method, *SC_intuit_*, to discriminate between the relative importance of poorly-conserved positions and well-conserved positions comparing it with the most representative scoring methods: *i) SC_indep_*, a probabilistic method that assumes positional independence; *ii) SC_dep_*, a scoring method proposed by Tomovic and Oakeley that take into account statistical interdependencies between TFBS positions [14]; and *iii) SC_mat_*, a scoring function proposed by Zare-Mirakabad et al. based on the dependency between all pairwise combinations of binding site positions [15].

**Figure 1:**
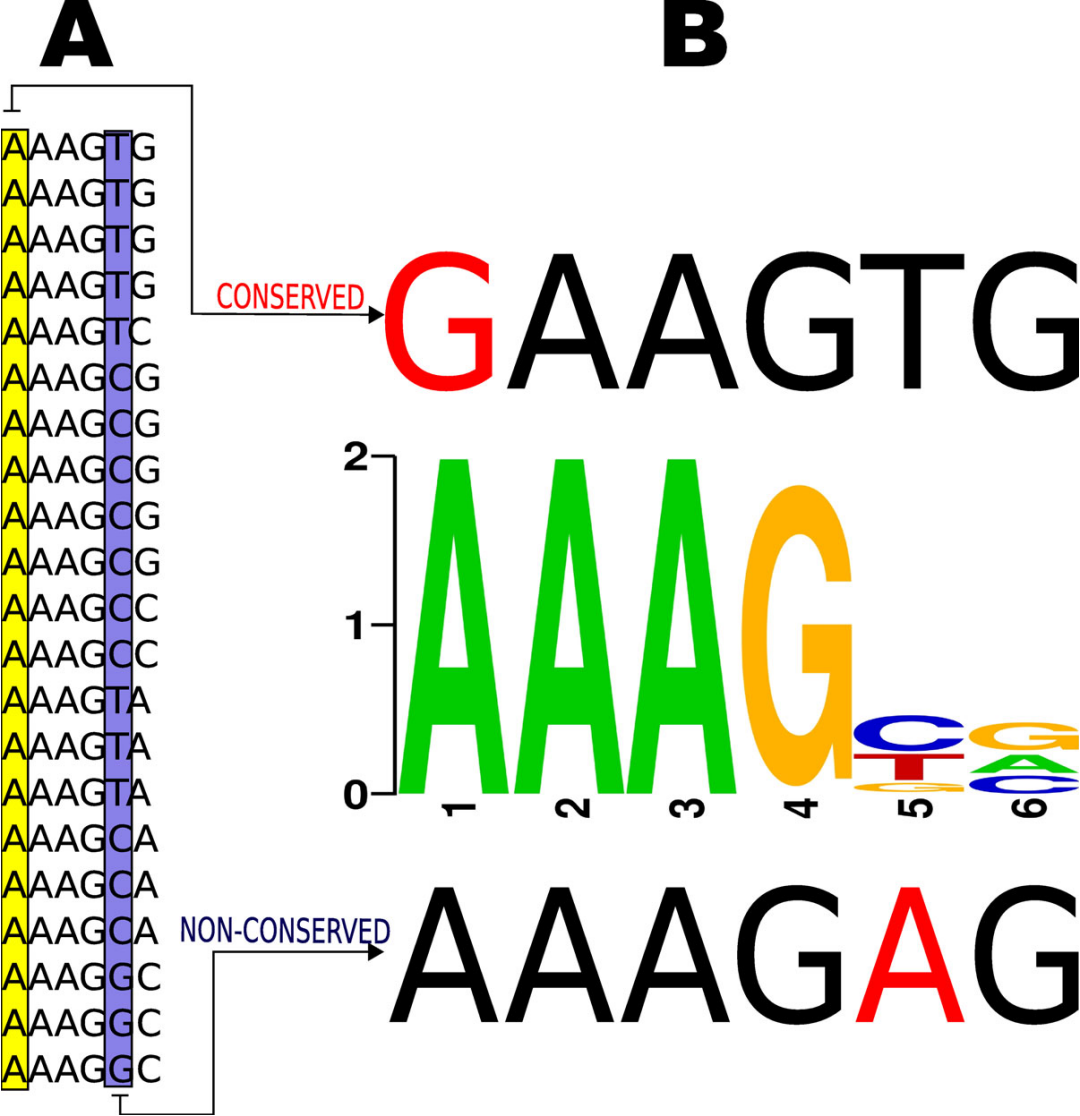
**Interdependencies between TFBS positions**. Motif Dof3. A) Shows the binding sequences found in JASPAR. B) Shows the logo representation of the motif (center), with the sequence having the mismatch in the conserved position (above), and the sequence having the mismatch in the poorly-conserved position (below).

In Figure 1(A) we show the binding sequences of the motif Dof3 found in the JASPAR database. It can be observed how the first position is highly conserved while the fifth position is poorly conserved. We then considered two sequences (Figure 1(B)): *i) *a sequence with a mismatch in the conserved position; and *ii) *a sequence with a mismatch in the poorly-conserved position. As has been explained above, it would be desirable that the score obtained for the case of the mismatch in the conserved position is lower than the scoring for the other sequence, as it shares the similarities in the most conserved positions of the motif. In Table 1 we show the results for the three methods. It can be observed that our proposed scoring method discriminates between the two cases, while the other three approaches provide almost the same score for both sequences, missing the difference between the conservation level of the positions being compared.

**Table 1:** Scoring results

	Non-conserved	Conserved	Difference
*SC_intuit_*	0.788	0.687	0.101
*SC_dep_*	0.832	0.815	0.017
*SC_mat_*	0.672	0.685	-0.013
*SC_indep_*	0.839	0.827	0.012

Scoring results for the first case study. Unlike the other approaches, *SC_intuit _*discriminates between the two sequences.

**Figure 2:**
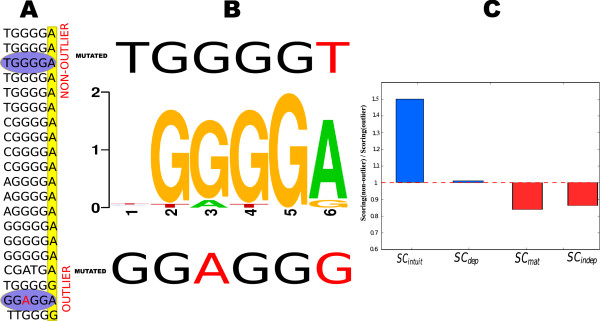
**Ratio of scoring values**. Motif MZF1. A) Shows the binding sequences found in JASPAR. B) Shows the logo representation of the motif (center), with the mutated sequences for the non-outlier binding sequence (above), and the outlier binding sequence (below). C) Shows the ratio of the scoring results for each of the three methods when scoring the mutated sequence of the non-outlier binding sequence and the outlier binding sequence respectively.

In the majority of cases, the sequences known to belong to a given TFBS motif have very similar nucleotide compositions and highly conserved positions. However, in the databases of known motifs there are a number of examples where individual sequences differ from the majority in highly-conserved positions. Such a binding sequence can be considered an outlier with respect to the motif, i.e. a binding site that is not closely related to the other binding sites in the motif. When scoring new sequences against a given TFBS motif, we should generally tolerate small, additional variations in the sequence with respect to non-outliers, but be far less tolerant of mutations to outlier sequences. Here we evaluate the extent to which each scoring method is able to discriminate between sequences belonging to these two categories. Take, as a preliminary example, the binding sequences for motif MZF1 in the JASPAR database, as shown in Figure 2(A). It can be observed how the highlighted outlier sequence GGAGGA does not contain the higly-conserved base G at the third position, while the highlighted sequence TGGGGA is clearly a non-outlier (see motif logo in Figure 2(B)). We selected the highlighted sequences and created two new sequences by mutating its sixth position giving GGAGGG (derived from an outlier) and TGGGGT (derived from a non-outlier). In order to observe the discrimination degrees of the different scoring methods, we scored each sequence against the motif by means of the different methods.

In reality, it would be desirable that the scoring for the case of the mutated outlier sequence be lower than the scoring for the mutated non-oulier sequence. Results obtained by the *SC_mat_*, *SC_dep_*, and *SC_indep _*methods failed to capture the expected differences, giving the incorrect impression that binding is likely to occur. On the other hand, our proposed method obtained a more realistic distance between the sequences, providing a much lower score for the mutated outlier sequence (Figure 2(C)).

These insights are confirmed in the following sections where the experiments are extended to use large datasets, and the results are measured in terms of discovery rates.

### Prediction of TFBSs

#### Synthetic sequences

In order to compare the performance of the different methods in predicting TFBSs, we used the non-redundant publicly available JASPAR motifs database for our experiments [23]. We selected all motifs for which binding sequences are available (not only matrix profiles), resulting in a dataset of 124 motifs. For each of these motifs, a random number between 2 and 6 binding sites were randomly selected and inserted in random sequences of a random length between 200 bp and 500 bp from a third-order Markov model background distribution obtained from the RSAT (Regulatory Sequence Analysis Tools) [24]. For each position of each sequence we computed the score for their corresponding motifs with an assumed known TFBS length (the length of the inserted motif).

Usually, methods have a high sensitivity (i.e. can detect true positives), so that the key difference between them is the number of false positives. Although our ultimate aim is not to rely on essentially arbitrary thresholds to assess performance, we began our analysis by following the recommendations of Tomovic and Oakeley in [14], selecting thresholds of 0.7 and 0.8 indicating a correct classification for a binding site. Table 2 shows the precision (*TP/*(*TP *+ *FP *)) of the different methods. In the additional file 1: "Synthetic sequences experiment" we show thresholded results for the different methods. These indicate that our proposed scoring function performed best, giving the smallest number of false positives per TF whilst simultaneously giving a high number of true positives.

**Table 2:** Synthetic sequences precision.

Threshold	***SC***_***intuit***_	***SC***_***dep***_	***SC***_***mat***_	***SC***_***indep***_
0.7	0.63	0.17	0.09	0.02
0.8	0.82	0.27	0.14	0.05

*SC_intuit _*shows a much better precision than the rest of the methods.

**Figure 3:**
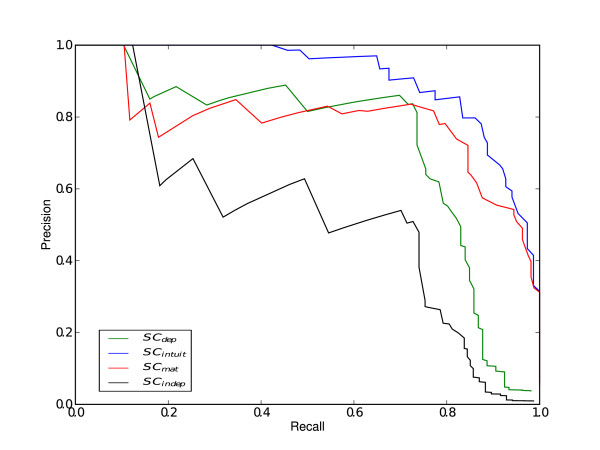
**Synthetic sequences results**. PR curves for the three scoring methods. *SC_intuit _*provides a more consistent classification than the rest of the methods.

In order not to rely on the selection of an arbitrary threshold for evaluating the results, we computed a precision-recall (PR) curve for each considered method. PR curves are commonly used in information retrieval for evaluating classification performance and give a more informative picture of a method's performance than ROC (Receiver Operating Characteristic) curves [25] when dealing with highly skewed datasets as is the case here [26]. Figure 3 shows the PR graphs. *SC_intuit _*produces a better PR graph than the remaining methods (see Table 3 for AUC values). In addition, ROC curves can be found in the the additional file 2: "ROC curves".

**Table 3:** AUC values for the synthetic and mutated sequence experiments.

	**Synthetic**	**Mutated**
		
*SC_indep_*	0.550	0.526
*SC_dep_*	0.730	0.705
*SC_mat_*	0.787	0.725
*SC_intuit_*	0.910	0.886

*SC_intuit _*provides better results for both experiments.

#### Mutated sequences

**Figure 4:**
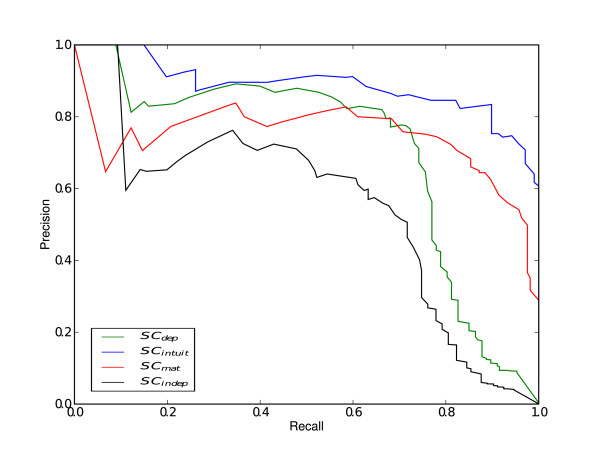
**Mutated sequences results**. PR curves for the three scoring methods. Again, *SC_intuit _*provides a better classification than the rest of the methods.

To further evaluate our proposed method, we obtained a set of putative binding sites that are very similar to those that are already known. This is a common scenario in motif discovery, where the set of known sequences belonging to a given binding motif is incomplete. In order to simulate this situation, we proceeded in a similar way to our previous experiment; all the steps were the same except that we gave a single base mutation at a random position within the selected binding site for each motif. PR curves and AUC values were computed to compare the performance of the different methods (Figure 4 and Table 3). ROC curves for this experiment can be obtained from the additional file 2: "ROC curves". The ROC and precision-recall graphs shows how *SC_intuit _*gives consistently superior values, with a higher AUC value (Table 3). It can be observed that the improvement of the performance of our method compared to *SC_indep_*, *SC_mat _*and *SC_dep _*grew with respect to the synthetic sequences experiment discussed in the previous section.

#### Real Data

**Figure 5:**
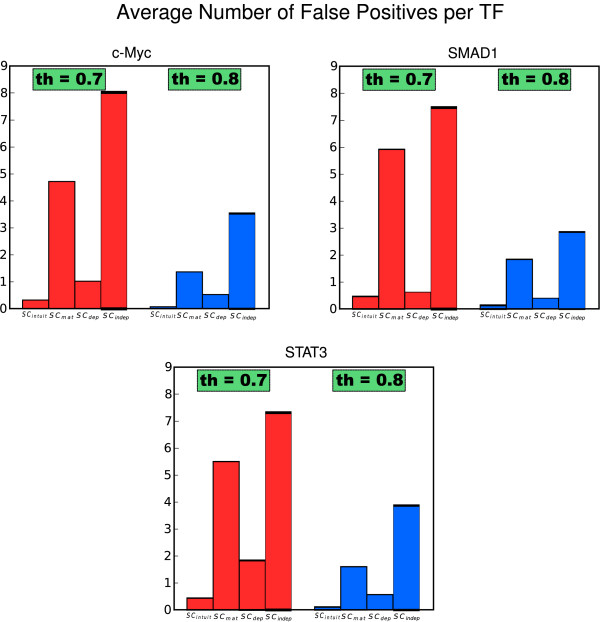
**Real data results**. Average false-positive ratio per TF for different thresholds for the proposed scoring methods.

We analyzed the performance of the proposed methods when dealing with real experimental data. In order to do so, we made use of the published ChIP-seq data on binding of TFs in embryonic stem cells from mouse by Chen et al. in [27], as provided in the supplementary material of [28]. We considered the three TFs (SMAD1, c-Myc, and STAT3) that have binding sequences available in the TRANSFAC database [29]. Thus, we obtained three sets of 200 bp sequence segments centered at TF binding locations, and we randomly selected 50 sequence segments from each set for our study (see additional file 3: "FASTA sequences"). We scanned each set of sequences using the 124 TFs from JASPAR for which binding sequences are available. The results demonstrate the superior performance of our new scoring method, as it gives the smallest number of false positives per nucleotide and per TF (Figure 5), and maintains an excellent true-positive rate (Table 4). Detailed results can be found in the additional file 4: "Motif statistics". It can be seen that our method presents consistently low false-positive rates with all three sets of sequences, whereas the performance of the other methods is much more variable.

**Table 4:** True positive rate for the real data experiment

TF	***SC***_***intuit***_	***SC***_***dep***_	***SC***_***mat***_	***SC***_***indep***_
SMAD1	0.96	0.94	0.90	0.86
c-Myc	0.92	0.94	0.92	0.84
STAT3	0.98	0.92	0.96	0.88

All the methods have a high true positive rate (threshold = 0.8).

### Study of Single Nucleotide Polymorphisms in TNFR1 Gene for the Response against Aspergillus Fumigatus

Hematological patients are typically treated by chemotherapy and/or radiation. These treatments usually produce immunosuppression and severe neutropenia. This clinical situation can be exploited by opportunistic pathogens such as *Aspergillus fumigatus *to cause a deadly infection called Invasive Pulmonary Aspergillosis (IPA) [30,31]. The importance of finding ways to combat this pathogen is evidenced by the fact that IPA occurs in roughly 10% to 40% of hematological patients, with overall mortality rates ranging from 50% to 90% [32,33].

Tumor necrosis factor (TNF) activates T lymphocytes in response to fungal infections through TNF receptors. One of the most important TNF receptors is TNFR1, which plays a crucial role in immune regulation and host immune responses. Experimental studies with TNFR1 knockout mice indicate that TNFR1 is indispensable in host resistance against several infections [34]. Our hypothesis is that single nucleotide polymorphisms (SNPs) in the TNFR1 gene may influence the innate immune response against *Aspergillus fumigatus*.

The gene encoding TNFR1 contain numerous polymorphisms [35,36]. By means of different experiments, we concluded that TNFR1_-609(G/T) _polymorphism is critical in the development of the response against *Aspergillus *because it might be regulating the cell-mediated Th1 immune response. Details on these experiments are out of the scope of this work and can be consulted in [37]. In this section, we use our proposed scoring method *SC_intuit _*to investigate whether the TNFR1_-609(G/T) _promoter polymorphism is involved in the disruption of the recognition of a potential binding site for a critical transcription factor that could influence TNFR1 transcription level.

#### TNFR1_-609(G/T) _Polymorphism Binding Affinity

For this experiment we used TFBS motifs found in TRANSFAC database [29], which has been widely used in research involving regulatory elements [38]. In order to find interesting dependencies between the TNFR1_-609(G/T) _SNP and TFs binding affinity we scored the human TRANSFAC TFBSs against the TNFR1_-609(G/T) _polymorphism by means of the *SC_intuit _*method.

**Figure 6:**
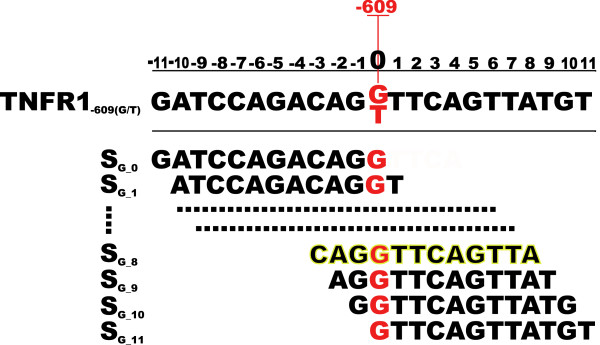
**Putative binding sequences for the G allele of the TNFR1_-609(G/T)_**. Best results were found for the highlighted S_*G*-8 _sequence. A corresponding set was also obtained for the T allele.

TFs bind to short parts of the TNFR1 promoter region and, therefore, for each trial, we need to define a fragment of the promoter sequence containing the TNFR1_-609(G/T) _SNP that might be considered as the putative TFBS. To this end, we need to determine the length of the sub-sequences and the relative offset to the position of the TNFR1_-609(G/T) _SNP. For each of the 446 human TFs in TRANSFAC, we generated a set of putative binding sequences by using a window size of a fixed length equal to the number of position of the corresponding TF. Moving the window across the sequence in 5'-3' direction gave us the sub-sequences for the TNFR1_-609(G/T) _SNP that we considered to be putative TFBSs (see Figure 6 for an example). Next, we scored each pair of sub-sequences (one sub-sequence for the G allele, and one for the T allele) against the given TF applying the *SC_intuit _*method.

We were interested in those sub-sequences that fulfil two properties: *i) *they have a high score in one allele (G or T) so they can be considered as candidates to be binding sites, and *ii) *the score is substantially lower when considering the remaining allele so the SNP may affect to the binding affinity. For our current research we chose a conservative cut-off of 0.7, and retained TFBSs with a score above this threshold for further analysis. The scores for the selected TFBSs with respect to their corresponding alleles (sequences with a G(T) instead of a T(G) at position -609) are shown in Table 5. Subsequently we will discuss these findings from a biological perspective and show that the most interesting insights arise in the context of the ICSBP TF, which represents the highest scoring of all the human TRANSFAC motifs.

**Table 5:** *SC*_*intuit *_scores for the two alleles.

TF	Starting position	Direction	**TNFR1-**_**609(T)**_	**TNFR1-**_**609(G)**_
AREB6	603	-	0.59	0,70
E2A	606	-	0.64	0.79
HNF4	605	+	0.52	0.78
**ICSBP**	606	+	**0.81**	**0.69**
MYB	601	-	0.76	0.77
Pax-2	604	-	0.76	0.58
SMAD	603	+	0.73	0.73

Human TRANSFAC motifs with a score greater than 0.7. ICSBP presents the highest scoring among all the human TRANSFAC motifs, showing a preference for binding the T allele.

#### Functional Effect of ICSBP/IRF-8 in the TNFR1_-609(C/T) _SNP

**Figure 7:**
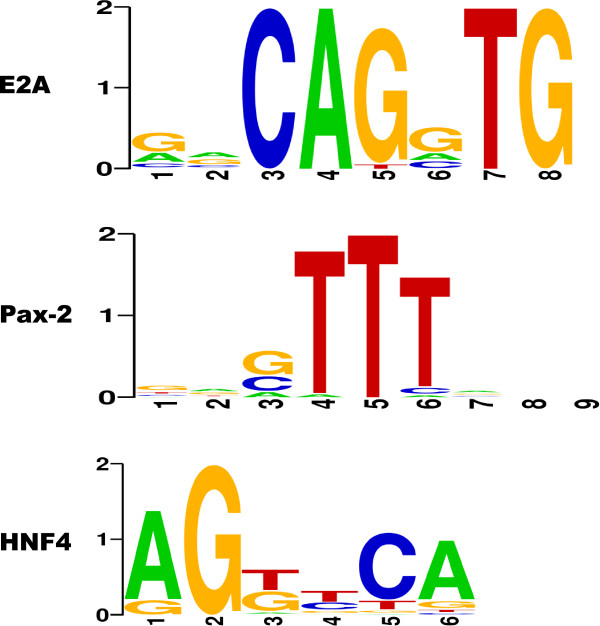
**Discarded TFs**. No described relations with IPA response were found.

In the previous section, we obtained predictive results using our *SC_intuit _*scoring method and TRANSFAC database (Table 5). From them, we selected four candidates according to the two properties outlined in the previous section, i.e E2A, HNF4, ICSBP, and Pax-2. We did not find described relations between IPA response for any of E2A, HNF4, and Pax-2 TFs. Logos for these TFs are provided in Figure 7.

On the other hand, we found ICSBP (also known as IRF-8) to be directly related with the purpose of our study. ICSBP/IRF-8 shows a preference for binding the T allele (see Table 5). As a member of IRF family of transcription factors it is an important modulator of IFNγ signalling cascade and was identified in association on the promoter region of numerous macrophage essential genes such as IL12, IL1*β*, IL18, iNOS or ISG15 [39].

In addition, several genes regulated by ICSBP/IRF-8, such as MAP4K4, IL-17R, and SOCS7, are involved in different stages of the nuclear factor *κ*B (NF*κ*B) signaling pathway [39]. Therefore, we can hypothesize that ICSBP/IRF-8 transcription factor might be also regulating the NF*κ*B signaling pathway through the control of the first gene of this signalling cascade, the TNFR1 gene. In support of this hypothesis, Zhao et al. established that ICSBP/IRF-8 and TNFR1 are closely related genes [40]. They found ICSBP/IRF-8 to be associated with an enhanced ubiquination of TNFR associated factor 6 (TRAF6), a protein that mediate the signal transduction from members of the TNF receptor superfamily, and the activation of AP-1 and NF*κ*B transcription factors.

On the other hand, several studies demonstrated that ICSBP/IRF-8 promotes the differentiation and activation of dendritic cells and macrophages cells [41,42], and that, at the same time, TNFR1 mRNA level is increased during this biological process [43].

**Figure 8:**
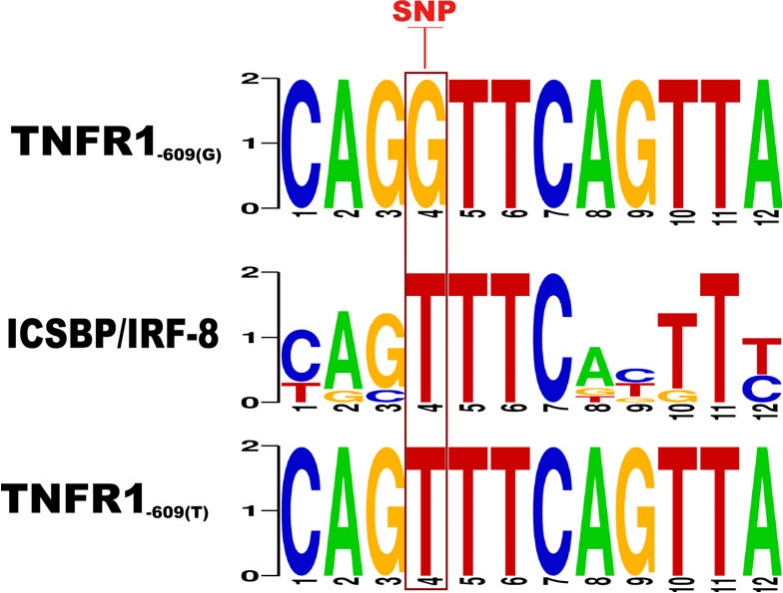
**ICSBP against TNFR1_-609(G/T) _polymorphism**. It can be seen how the fourth position in the ICSBP motif (middle) matches the T allele (bottom) and mismatches the G allele (top).

Taken into account these observations, we hypothesize that the presence of TNFR1_-609(G/T) _promoter polymorphisms can modify the binding affinity to ICSBP/IRF-8 (see Figure 8) and, therefore, it could be used to predict susceptibility to infection and to facilitate risk stratification of hematological patients. However, the question of whether the TNFR1 polymorphisms have biological relevance regulating mRNA TNFR1 levels through ICSBP/IRF-8 transcription factor remains unanswered. Functional analysis should be performed to demonstrate the role of TNFR1_-609(G/T) _polymorphism mediating the binding of ICSBP/IRF-8 to TNFR1 promoter.

## Discussion

We have introduced a new IFS-based approach for scoring DNA sequences against DNA motifs called *SC_intuit_*. In this work we review three scoring schemes. These approaches have several drawbacks. *SC_indep _*is based on an incorrect assumption that the nucleotides of a given TFBS are independent. In that context, *SC_dep _*extended the score in order to account for positional dependencies. The problems associated with unnormalized scores at each position have been pointed out [15]. In addition, the results vary depending on the choice of the method and parameters for testing the dependencies. The main drawback with *SC_mat _*is that it has a tendency to overlearn the training data and consequently its performance decreases when applied to real problems. There is therefore a need for a scoring method that accounts for positional dependencies without compromising either the consistency or the accuracy of the results.

As explained above, *SC_intuit _*is based on the IFS theory, which has been successfully applied to problems that suffers from noisy and imprecise data. IFS theory represents uncertainty with respect to both the degree of membership and non-membership. The uncertainty associated with the tasks of scoring DNA sequences against motifs makes intuitionistic concepts particularly suitable for handling this kind of data. Taking advantage of such properties, we define the membership and non-membership degrees of a given pair bases at a given position not only as a function of their combined probability of occurrence, but also taking into account the importance of each individual base at its corresponding position.

One of the biggest issues for this kind of scoring methods is giving high scores for the known binding sequences of the motifs without overfitting. Our proposed approach adequately solves the problem of computing the score of a given sequence against a given motif by considering the binding sequences that comprise the motif not only individually but also as part of such set of sequences. Simple experiments shows how other methods fail in capturing realistic differences, while *SC_intuit _*provides good results (Figure 1, 2). Our method assigned high scores for known binding sites, disfavouring mutations in the conserved positions of the binding site.

These insights are confirmed from experiments for predicting TFBSs in large datasets. We compared the performance of the proposed scoring methods on recognizing motifs in sets of random sequences from a third-order Markov model background distribution in two circumstances: *i) *when inserting known binding sequences, and *ii) *when inserting mutated binding sequences. In both situations we found that our proposed method gave the smallest number of false positives per TF whilst simultaneously giving a high number of true positives (Figures 3, 4). More importantly, our method outperforms the other approaches when dealing with real experimental data derived from Chip-seq assays. In this case, again, the number of false positive is significantly reduced (Figure 5). Finally, we validated our method studying the effect of known SNPs of TNFR1 gene in the binding affinity of TRANSFAC TFs for the response against Aspergillus fumigatus. We found the highest scoring for ICSBP TF among all the human TRANSFAC motifs. Although functional analysis should be performed, according to several previous studies, we hypothesize that the presence of TNFR1_-609(*G/T*) _polymorphisms could be used to predict susceptibility to infection of hematological patients.

In general, the obtained results on the different experiments demonstrated that the proposed intuitionistic approach provide a better and more accurate model for the detection of motifs and for the relationships between positions of the TFBSs.

## Conclusions

In the present study, we have introduced *SC_intuit_*, a new scoring method for measuring sequence-motif affinity, based on IFS theory. Our main objective was to improve the prediction quality for TFs of the existing approaches, reducing the false positive rate without compromising sensitivity. We show that *SC_intuit _*outperforms other approaches in motif recognition tasks, and prove how it can be successfully applied to real research problems. We have used our approach as a scanning method for the prediction of TFBSs, but it also can be incorporated with methods for *de novo *discovery of motifs. As intuitionistic theory is specially suitable for problems that deal with imprecise concepts, we are currently working on a fuzzy approach that applies the proposed scoring in an *ab initio *method to find motifs in large sets of related DNA sequences.

## Methods

### Alternative approaches

In recent years, several scoring methods for the prediction of TFBSs have been proposed. In this section we give a brief overview of those methods that take account of positional dependencies, as they have been shown to outperform methods that assume independence. Let us first introduce the notation. Let *B *= {*A*, *C*, *G*, *T*} be the set of the four DNA nucleotides. Let *D *be a set of ordered DNA sequences on *B *of length *n*. Let us suppose that we have a motif *M *= *S*_1_,..., *S_t_*, where *S_i _*is a DNA sequence on *D *consisting of *t *aligned binding sites of length *n*. The problem is then reduced to assigning a score to the pair formed by a given putative TFBS, *S *∈ *D*, and a given motif, *M*.

In what follows we will follow the notation proposed by Wasserman and Sandelin in [44], where *F *(*b*, *i*), for *b *∈ *B *and 1 ≤ *i *≤ *n *shows the occurrences of nucleotide *b *in position *i*, and $P(b,i)=\frac{F(b,i)}{t}+a(b)$, for *b*∈ * B *and 1 ≤ *i *≤ *n *is the corrected probability of base *b *at position *i*, where *a*(*b*) is a smoothing parameter (*a*(*b*) = 0.001). *a*(*b*) = 0.01 is usually reported but our experiments show that smaller values provide more accurate results.

#### Statistical dependencies

Tomovic and Oakeley extended the previous method that assumed positional independence [14]. The authors also followed the notation of [44] and defined *W*_*b*, *i *_as a position weighted matrix (PWM) of base *b *in position *i *computed as:

(1) $$W_{b,i}=log_{2}\frac{P(b,i)}{P(b)},$$

where *P*(*b*) is the background probability of base *b*. In the case where independence is assumed, the score for a given DNA sequence *S *can be computed by summing all the values of *W*_*b*, *i *_for every base in *S*:

(2) $$SC_{indep}(s)=\sum_{i=1}^{n}W_{s_{i},i}.$$

The first step for extending this score involves testing the dependencies between each pair of positions *i *and *j*. The authors introduced three different methods: *i*) χ^2 ^test; *ii*) *G *statistics; and *iii*) Bayesian hypothesis testing. The authors used these three methods to calculate the dependencies between pairs of positions in the motifs available in the public database JASPAR [23]. The reader should note that the accurate computation of positional dependencies is still an open problem since different results are obtained depending on the method and parameters used in their computation (see Supplementary Material 2-4 in [14]). Further details about obtaining the position dependencies and multiple test corrections can be found in [14].

In order to compute the new score, the corrected probability for the bases *b*_1_*b*_2 _... *b*_*m *_in the dependent positions *i*_1_*i*_2 _... *i*_*m *_is defined by:

(3) P(b1,…,bm,i1,…,im)=F(b1,…,bm,i1,…,im)t++a(b1,…,bm),

where *a*(*b*_1_,..., *b_m_*) = *a*(*b*_1_) ... *a*(*b_m_*) is a smoothing parameter.

It is straightforward then to obtain values that correspond to the PWM values:

(4) $$W_{b_{1},\ldots ,b_{m},i_{1},\ldots ,i_{m}}=\log_{2}\left(\frac{P(b_{1},\ldots ,b_{m},i_{1},\ldots ,i_{m})}{P(b_{1})\ldots P(b_{m})}\right).$$

Finally, their proposed scoring function, which incorporates positional dependencies, can be computed as:

(5) SCdep(S)=∑i=1k1WSi,i+∑i=1k2WSji,Sji+1,ji,ji+1+⋯++∑i=1kmWSji,…,Sji+m−1,ji,…,ji+m−1,

where, *k*_1 _is the number of independent positions, *k*_2 _is the number of dependent positions of order 2 (nucleotides at positions *j*_*i *_and *j*_*i*+1_) and *k*_*m *_the number of dependent positions of order m (nucleotides at positions *j*_*i*_, *j*_*i*+1_,..., *j*_*i*+*m*-1_).

For both the *SC_indep _*and *SC_dep _*it is advisable to perform the following normalization:

(6) $$N_{SC}=\frac{SC-\min(SC)}{\max(SC)-\min(SC)}$$

#### Matrix based

Zare-Mirakabad et al. proposed a new scoring function based on the dependency between all pairwise combinations of binding site positions [15]. Their method is based on the mutual information matrix, defined as: (see equation (7))

(7) $$M_{ij}=\sum_{b_{i},b_{j}}P(b_{i},b_{j},i,j)\log_{2}\left(\frac{P(b_{i},b_{j},i,j)}{P(b_{i},i)P(b_{j},j)}\right),$$

and on the joint information content (JIC), defined as:

(8) $$JIC=\sum_{i=1}^{n-1}\sum_{j=i+1}^{n}\sum_{{}_{b_{1}\in B}}\sum_{{}_{b_{2}\in B}}P(b_{1},b_{2},i,j)\log\left(\frac{P(b_{1},b_{2},i,j)}{P(b_{1})P(b_{2})}\right).$$

In order to compute their score, the authors defined a PWM, *W^PW ^*, containing 16 rows and (*n *· (*n *- 1)/2) columns for all the pairwise combinations of the positions:

(9) $$W_{b_{1},b_{2},i,j}^{PW}=\log\left(\frac{P(b_{1},b_{2},i,j)}{P(b_{1})P(b_{2})}\right)+\log\left(\frac{P(b_{1},b_{2},i,j)}{P(b_{1},i)P(b_{2},j)}\right),$$

where *b*_1, _*b*_2 _∈ *2 B *and 1 ≤ i, *j *≤ *n *and *i *≠ *j*. For more on this method see [15].

Finally, for a given DNA sequence *S *∈ *D *of length *n *the score *SC_mat _*is computed as:

(10) $$SC_{mat}=\sum_{i=1}^{n-1}\sum_{j=i+1}^{n}W_{S_{i},S_{j},i,j}^{PW}.$$

In order to obtain a normalized value for the score, equation (6) should be applied.

### Intuitionistic fuzzy sets

Intuitionistic fuzzy sets (IFS) theory was proposed by Atanassov [17]. It has been applied in such diverse fields as decision making [45], logic programming [46] medical diagnosis [47,48], pattern recognition [49], etc. IFS theory is an extension of the fuzzy sets theory previously proposed by Zadeh [16] that allows the degrees of membership and non-membership to be independently uncertain, which makes the representation more flexible at capturing the current state of our understanding given inconclusive data [50,51]. Next, we introduce some basic IFS concepts.

Let *X *be the universe of discourse. An intuitionistic fuzzy set *A *in *X *is an object having the form:

(11) $$A=\{(x,\mu_{A}(x),\nu_{A}(x)):x\in X\},$$

where *μ*_*A*_, *ν*_*A *_: *X *→ 0[1] denote membership function and non-membership function of *A*, satisfying 0 ≤ *μ_A _*+ *ν*_*A *_≤ 1 for every *x *∈ *X*. Therefore, the degree of uncertainty of *x *to *A *is *π*_*A*_(*x*) = 1 - *μ_A _*- *ν*_*A*_. For more on this topic please refer to [17,50,51].

### Intuitionistic representation of motifs

For our approach, a given motif *M *is represented as the set of IFSs of all the pairwise combinations of its positions: $I^{M}=\{I_{i,j}^{M}\}$, where 1 ≤ *i*, *j *≤ *n *and *i *≠ *j*. Each of the *i*, *j *combinations for the motif positions is then an IFS of 16 elements defined as:

(12) $$I_{i,j}^{M}=\{b,\mu_{I_{i,j}^{M}}(b),v_{I_{i,j}^{M}}(b):b\in B\times B\},$$

where *B *× *B *is the universe of discourse, i.e. the set of all 16 possible combinations of bases for two given positions *i *and *j *(AA, AC,..., TT).

#### Membership degree computation

$\mu_{I_{l,j}^{M}}$ represents the degree of membership of the pairs for the basis *b*_1, _*b*_2 _∈ *B *in a given pair of positions *i*, *j *in a motif *M*. It can be automatically computed as:

(13) $$\mu_{I_{i,j}^{M}}(b_{1},b_{2})=P(b_{1},b_{2},i,j)+(1-P(b_{1},b_{2},i,j))\frac{P(b_{1},i)+P(b_{2},j)}{2},$$

where the above notation holds. As can be seen, the membership degree is a function of the probability of the pair of bases being compared and their individual conservation. Obviously, $0\leq \mu_{I_{i,j}^{M}}(b_{1},\;b_{2})\leq 1$ and the degree increases as do the corrected probabilities of bases *b*_1 _and *b*_2 _in positions *i *and *j*, as well as the individual corrected probabilities *P *(*b*_1, _*i*) and *P *(*b*_2, _*j*).

#### Non-membership degree computation

$\nu_{I_{i,j}^{M}}$ represents the non-membership degree of the pairs for the basis *b*_1, _*b*_2 _∈ *B *in a given pair of positions *i *and *j *in a motif *M*. It can be automatically computed as:

(14) $$\nu_{I_{i,j}^{M}}(b_{1},b_{2})=\left(\frac{IC_{i}^{b_{1}}+IC_{j}^{b_{2}}}{2}\right)(1-\mu_{I_{i,j}^{M}}(b_{1},\;b_{2})),$$

where $IC_{p}^{b}=\frac{2+P(b,p)\log_{2}(P(b,p))}{2}$ is the normalized information content of base *b *in position *p *and $\nu_{I_{i,j}^{M}}(b_{1},\;b_{2})$ is in the range $0\leq \nu_{I_{i,j}^{M}}(b_{1},\;b_{2})\leq 1$. The reader should note that we make use of the IC of the two positions *i*, *j *to assign the allowed degree of uncertainty for such pair of positions. When the IC is high, the degree of uncertainty will be low and viceversa. Likewise, as the information content of the two basis increases, the sum of the membership degrees, $\mu_{I_{i,j}^{M}}(b_{1},\;b_{2})+\nu_{I_{i,j}^{M}}(b_{1},\;b_{2})$, gets closer to 1. Therefore, the non-membership degree in positions *i*, *j *is a function of the corresponding membership degree and the uncertainty level computed for *b*_1 _and *b*_2_. It is easy to prove that $\mu_{I_{i,j}^{M}}(b_{1},\;b_{2})+\nu_{I_{i,j}^{M}}(b_{1},\;b_{2})\leq 1$

### Scoring

In order to define our proposed score, we first introduce the simplest case of scoring a length-2 DNA subsequence *D *= *b*_1_, *b*_2 _in the positions *i *and *j *of a motif *M*:

(15) $$SC_{intuit}^{i,j}(b_{1},b_{2})=\mu_{I_{i,j}^{M}}(b_{1},b_{2})(\max(\nu_{I_{i,j}^{M}})-\nu_{I_{i,j}^{M}}(b_{1},b_{2})),$$

where $\max(\nu_{I_{i,j}^{M}})$ is the maximum degree of non-membership in M found in the pair of positions *i *and *j *considering all the possible combination of basis *b*_1_, *b*_2 _∈ *B*^2^, and $\mu_{I_{i,j}^{M}}(b_{1},\;b_{2})$ and $\nu_{I_{i,j}^{M}}(b_{1},\;b_{2})$ are the membership degree and non-membership degree of the pairs for the basis (*b*_1_, *b*_2_) ∈ *B *in the pair of positions *i*, *j *of *M*, computed as stated in sections and respectively.

As with the previously defined scores, a normalization step needs to be performed in order to obtain comparable results. The source code can obtained from http://genome.ugr.es/intuit.

(16) $$NSC_{intuit}^{i,j}(b_{1},b_{2})=\frac{SC_{intuit}^{i,j}(b_{1},b_{2})-\min(SC_{intuit}^{i,j})}{\max(SC_{intuit}^{i,j})-\min(SC_{intuit}^{i,j})},$$

where are the $\min(SC_{intuit}^{i,j})$ and $\text{max}(SC_{intuit}^{i,j})$ are the min/max possible scores in the positions (*i*, *j*) of the motif.

Finally, for a given DNA sequence *S *∈ *D *of length *n *the score *SC_intuit _*is computed as:

(17) $$SC_{intuit}=\sum_{i=1}^{n-1}\sum_{j=i+1}^{n}NSC_{intuit}^{i,j}(S_{i},S_{j}).$$

## Authors' contributions

FG designed the study, designed and implemented the intuitionistic scoring, performed the experiments, helped with the analysis of the results and drafted the paper. AB assisted with the design of the study and helped to draft the paper. AS provided help with the analysis of the results and assisted in drafting the paper. All authors read and approved the final manuscript.

## Supplementary Material

Additional file 1 **Synthetic sequences experiment**. This files contains thresholded results for the different methods for the synthetic sequences experiment. Click here for file

Additional file 2 **ROC curves**. This file contains the ROC curves associated to the synthetic and mutated sequences experiments. Click here for file

Additional file 3 **FASTA sequences**. This file contains the 50 sequence segments in FASTA format for each one of the motifs SMAD1, Myc, and STAT3. Click here for file

Additional file 4 **Motif statistics**. This file contains some statistics for the experiment discussed in the section *Real data*. Click here for file
