# Supplementary material for: An intuitionistic approach to scoring DNA sequences against transcription factor binding site motifs
Source: BMC Bioinformatics. 2010 Nov 8;11:551. doi: 10.1186/1471-2105-11-551 (PMC3098096; doi:10.1186/1471-2105-11-551)
Supplement: Additional file 2 — ROC curves. This file contains the ROC curves associated to the synthetic and mutated sequences experiments. [file 1471-2105-11-551-S2.PDF]

Additional file 2 — ROC curves

1 Synthetic sequences

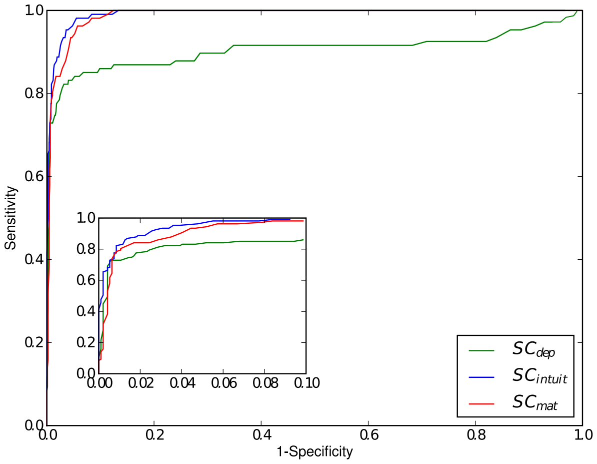

Figure 1: ROC curves for the synthetic sequences experiment.

2 Mutated sequences

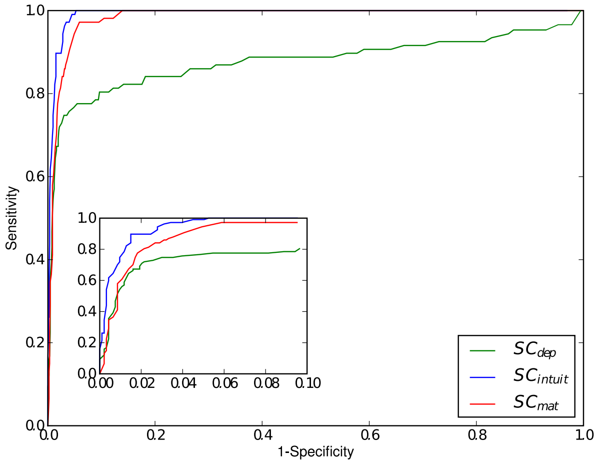

Figure 2: ROC curves for the mutated sequences experiment.
